# Promoting sustainability in quality improvement: an evaluation of a web-based continuing education program in blood pressure measurement

**DOI:** 10.1186/s12875-017-0682-5

**Published:** 2018-01-10

**Authors:** Lauren Block, Sarah J. Flynn, Lisa A. Cooper, Caroline Lentz, Tammie Hull, Katherine B. Dietz, Romsai T. Boonyasai

**Affiliations:** 1 Donald and Barbara Zucker School of Medicine, Hofstra/Northwell 500 Hofstra University, Hempstead, NY 11594 USA; 2 University of Maryland School of Medicine, 655 West Baltimore St, Baltimore, MD 21201 USA; 3 Johns Hopkins Center to Eliminate Cardiovascular Health Disparities, Department of Medicine, Johns Hopkins University School of Medicine, 2024 E. Monument St, 2-600, Baltimore, MD 21205 USA; 4 Johns Hopkins University Bloomberg School of Public Health, 615 N Wolfe St, Baltimore, MD 21205 USA; 5 Johns Hopkins Community Physicians, 3100 Wyman Park Dr, Baltimore, MD 21211 USA; 6 Division of General Internal Medicine, Northwell Health, 2001 Marcus Avenue, Suite S160, Lake Success, NY 11042 USA

**Keywords:** Blood pressure measurement, Sustainability, Web-based education, Continuing education, Quality improvement

## Abstract

**Background:**

The accuracy of blood pressure measurement is variable in office-based settings. Even when staff training programs are effective, knowledge and skills decay over time, supporting the need for ongoing staff training. We evaluated whether a web-based continuing education program in blood pressure measurement reinforced knowledge and skills among clinical staff and promoted sustainability of an existing quality improvement program.

**Methods:**

Medical assistants and nurses at six primary care clinics within a health system enrolled in a 30-min online educational program designed to refresh their knowledge of blood pressure measurement. A 20-question pre- and post-intervention survey addressed learners’ knowledge and attitudes. Direct observation of blood pressure measurement technique before and after the intervention was performed. Differences in responses to pre- and post-module knowledge and attitudes questions and in observation data were analyzed using chi-square tests and simple logistic regression.

**Results:**

All 88 clinical staff members participated in the program and completed the evaluation survey. Participants answered 80.6% of questions correctly before the module and 93.4% afterwards (*p* < 0.01). Scores improved significantly among staff from all job types. Licensed practical nurses and staff who had been in their current job at least a year were more likely to answer questions correctly than registered nurses and those in their current job less than a year. Attitudes toward correct blood pressure measurement were high at baseline and did not improve significantly. Prior to the intervention, staff adhered to 9 of 18 elements of the recommended technique during at least 90% of observations. Following the program, staff was more likely to explain the protocol, provide a rest period, measure an average blood pressure, and record the average blood pressure, but less likely to measure blood pressure with the arm at heart level and use the right arm.

**Conclusions:**

We designed, implemented, and evaluated a web-based educational program to improve knowledge, skills, and attitudes in blood pressure measurement and use of an automated device among nurses and medical assistants in ambulatory care. The program reinforced knowledge related to recommended blood pressure measurement technique.

**Trial registration:**

Retrospectively registered with ClincalTrials.gov on March 22, 2012; registration number NCT01566864.

**Electronic supplementary material:**

The online version of this article (10.1186/s12875-017-0682-5) contains supplementary material, which is available to authorized users.

## Background

Office-based blood pressure measurement is variable, even among clinical staff accustomed to measuring blood pressure [1–3]. Failure to adhere to American Heart Association (AHA) blood pressure measurement guidelines has been shown for practicing primary care physicians and nurses [2, 4]. Poor measurement technique can lead to both under- and over-treatment of hypertension, and may contribute to suboptimal control [4]. Lack of supervised training, knowledge of correct technique, and degradation of knowledge and skills have been cited as contributing factors [5–8]. Staff training programs may minimize measurement error, especially when integrated with the use of automated blood pressure devices [9, 10] as part of comprehensive quality improvement “bundles” [11, 12].

However, implementation and sustainability of quality improvement programs such as these may be hindered by factors such as lack of integration with existing workflow, degradation of knowledge or skills, lack of resources, and competing demands [13]. For example, in the area of blood pressure measurement, decay in staff knowledge and skills necessitates ongoing refresher training in order to ensure program sustainability [14]. Structured ongoing training in correct technique has been effective in improving knowledge and behaviors among clinical staff, but programs described in the literature have been conducted in-person over the course of several hours, consuming both staff and teaching faculty time [1, 5].

Sustainability, defined as “making an innovation routine until it reaches obsolescence,” may be conceptualized as depending on two conditions: institutionalisation and routinisation [15, 16]. *Institutionalisation* refers to the process by which organizations provide the resources and conditions needed to support a new practice. *Routinisation* refers to the process by which a new practice becomes a routine activity for workers within that organization. In other words, a program’s sustainability depends both on the extent to which an organization integrates a new practice into its physical infrastructure, policies, and management activities; and on workers developing a culture in which the new practice is treated as, “the way we do things here.” Although both conditions must be present to ensure a new practice’s sustainability, organizational leaders typically have more direct control over processes related to institutionalisation [16].

Web-based training has gained popularity as a modality for delivering up-to-date, standardized, accessible content that can be integrated with existing training systems, facilitating both institutionalisation across multiple sites and routinisation [17]. Web-based training can be disseminated across disparate care centers, offering the promise of delivering targeted, up-to-date training in line with organizational goals [18, 19]. As hospitals and physician practices continue to consolidate, the need for integrated, standardized training will only increase [20]. To our knowledge, no published studies of have addressed use of an online educational program towards sustainable quality improvement across a health system.

We designed, implemented, and evaluated a concise web-based educational program to improve knowledge, skills, and attitudes in blood pressure measurement and use of an automated device among nurses and medical assistants in ambulatory care. In this paper we report on the effectiveness as well as the institutionalisation of this intervention.

## Methods

### Context and setting

The study was conducted as part of Project ReD CHiP (Reducing Disparities and Controlling Hypertension in Primary Care), a pragmatic trial designed to evaluate multiple interventions for improving blood pressure control and reducing hypertension-related racial disparities conducted in six primary care practices within the Johns Hopkins Community Physicians (JHCP) health system [11].

In 2011, approximately 2 years prior to this study, we trained clinic staff to use the Omron HEM-907XL automated blood pressure measurement devices in a manner consistent with AHA guidelines [21]. As part of the practice network’s protocol, staff had been instructed to position patients in accordance with AHA guidelines, to rest patients for 3 min and then to obtain three consecutive blood pressure measurements using the automated features of the device [21]. The online training program described in this study was developed to address the organization’s need to train new employees and provide ongoing training for existing staff.

### Study design

We conducted a pre- and post-intervention assessment of knowledge, attitudes, and behaviors among JHCP staff who participated in this online training program. All certified medical assistants (CMAs), licensed practical nurses (LPNs), and registered nurses (RNs) at intervention practices were invited to enroll. Prior to enrolling in this study, all staff members had already been trained and deemed proficient in performing the ReD CHiP blood pressure measurement process, including use of the automated device, which had become the study clinics’ standard blood pressure measurement technique. Initial training at hire consisted of group didactics and individual tests of proficiency via direct observation by JCHP nurse educators.

### Ethics, consent, and permissions

This study was approved and HIPAA waiver granted by the Institutional Review Board at Johns Hopkins University School of Medicine (NA_00037622) and the Johns Hopkins Community Physicians Education Committee. As the procedures were classified by the IRB as comprising quality improvement endeavors, written consent was not obtained.

### Continuing education program

The training program consisted of two 15-min videos and 20 pre- and post-module multiple choice questions that were delivered via Course Development (LearnShare LLC, Maumee, Ohio), an online educational platform that was available to the entire health system. All questions were vetted by program leadership and the web development team. For this study, clinical staff members viewed the videos during a 1 month period between April–May, 2013 and were required to score ≥ 80% on knowledge questions to pass. Those who did not score at least 80% were required to re-watch the training modules and re-take the post-module test. Module topics included the rationale behind blood pressure measurement, patient positioning, importance of a rest period and taking multiple measurements, key features of the automated device, troubleshooting device errors, and measuring blood pressure in emergency settings and special populations such as obese and physically disabled patients. To promote peer validation, two CMAs from one of the practices demonstrated correct blood pressure measurement techniques in the training videos [13].

### Data collection

#### Knowledge and attitude outcomes

Knowledge and attitude outcomes were assessed using pre- and post-module questions, which consisted of a 15-question knowledge test and a 5-item survey of participants’ attitudes towards the blood pressure measurement program (Additional file 1). The knowledge test covered the importance of blood pressure control, patient positioning, rest period, and use of the automated device. Attitudes questions related to the importance of following AHA guidelines in all patients. A 5-point Likert scale from “1 – strongly agree” to “5 – strongly disagree” was used for responses to attitudes questions.

#### Behavior outcomes

To assess participants’ behavior, research assistants (RAs) observed a semi-random convenience sample of CMAs measuring blood pressure during the 4–8 weeks prior to and after the intervention, with a goal of observing between 2 and 3 patients per CMA at the clinical site. RAs were instructed to obtain observations during both morning and afternoon sessions throughout the week. RAs approached the first patient who presented to the clinic for registration. If the patient agreed to being observed, RAs collected demographic information and accompanied the patient to the exam room, where the RA recorded observations about blood pressure measurement using a structured form (Additional file 2) until the intake process ended. Information about the staff conducting the blood pressure measurement was not recorded. After completing an observation, RAs then approached the next patient presenting for registration and enrolled them if the next patient would be seen by a different CMA. To minimize risk for selection bias and for the Hawthorne effect, RAs were instructed to tell clinic staff that they were observing all aspects of the patient intake process instead of specifically blood pressure measurement. To establish the inter-rater reliability among observers, RAs rated four pre-recorded videos of mock office visits prior to deployment to the clinics. Concordance rates among the four RAs and three investigators (LB, SF, RB) was ≥ 85%.

#### Modifying variables

Staff participant demographic information was gathered from practice network administrative data, including gender, age, race, ethnicity, practice site, time in current job, and job title (CMA vs. LPN vs. RN). Variables for observer, site, and time of day also were recorded with direct observations.

#### Data analysis

Responses to knowledge questions were classified as correct or incorrect and tallied by individual participant and overall by pre- or post-module. For behavior outcomes, independent variables were observer and site. Dependent variables were aspects of blood pressure measurement. After the intervention, we learned that an incorrect response had been posted in the pre-assessment but not the post-assessment. The question, regarding patient positioning, gave the correct answer as “the patient’s arm should be held away from his/her body” rather than “the patient’s back should be supported during blood pressure measurement.” This question was therefore removed from data analysis.

We also conducted a post-hoc, secondary analysis to assess for links between when an education topic was delivered, duration of time allocated to that topic or quality of educational content, and staff members’ knowledge. To assess timing and duration of an educational topic, we coded the time when each aspect of blood pressure measurement technique was introduced, and the duration of time allocated to discussing a given technique. To assess quality of educational content, we first coded whether the module only verbally described an aspect of recommended blood pressure measurement technique or whether it also showed the underlying reason for why that aspect of technique is important. We also coded content on whether the module described a given aspect of technique verbally, visually, with written descriptors, or with a combination of these methods.

Differences in responses to pre- and post-module knowledge and attitudes questions and in observation data were analyzed using chi-square tests and simple logistic regression. STATA 11IC (Stata Corp, College Station, TX) was used to perform all analyses.

## Results

All 88 invited clinical staff completed the continuing education program including the pre- and post-test (100% response, Table 1). Ninety-eight percent of participants were female; 73% were younger than age 46. 72% were CMAs, 22% were RNs and 7% were LPNs. Most (52%) were in their current job at least 3 years. On average, module completion took 26.9 min.

**Table 1:** Characteristics of participants in the educational program

Characteristics	Participants *N*, (%)
Total	88 (100%)
Age, years	
18–30	26 (30%)
31–45	38 (43%)
46–60	21 (24%)
61+	3 (3%)
Male sex	2 (2%)
Race	
White	47 (53%)
African-American	32 (36%)
Asian	2 (2%)
Other	7 (7%)
Hispanic	3 (3%)
Profession^a^	
CMA	63 (63%)
RN	19 (22%)
LPN	6 (7%)
Practice site^b^	
Site C	25 (28%)
Site B	23 (26%)
Site A	18 (20%)
Site E	11 (13%)
Site D	6 (7%)
Site F	5 (6%)
Time at practice site	
Less than 1 year	15 (17%)
1–3 years	27 (31%)
More than 3 years	46 (52%)

^*a*^*CMA* certified medical assistant, *RN* registered nursem, *LPN* licensed practical nurse

^b^Description of these practice sites can be found in Cooper LA, et al., Implement Sci 2013[11]

### Knowledge and attitudes

Mean attitudes scores toward correct blood pressure measurement was positive at baseline (mean score 4.2 out of 5) and did not improve significantly post-module (mean score 4.3, *p* = 0.33) (Table 2, Additional file 3). Participants answered 80.6% of knowledge questions correctly before viewing the module and 93.4% of questions correctly after viewing the module (*p* < 0.01) (Table 1). Scores improved significantly among staff from all job types. LPNs and staff who had been in their current job at least a year were more likely to answer questions correctly than RNs (p < 0.01) and those in their current job less than a year, respectively (*p* = 0.04). Demographic factors including age, gender, race and ethnicity were not associated with higher scores. No baseline differences in knowledge were found between the 3 sites with predominantly underserved populations and the other 3 sites. No correlation was seen between time allocated to the learning objective in the video and score on the knowledge assessment. All learning objectives were provided using verbal plus visual and/or text-based instruction, and all except one provided the reasoning for the technique as well as the recommendation.

**Table 2:** Assessment of participants’ knowledge and attitudes, pre- and post-program, *N* = 88

	Pre-module responses, *N* (%)	Post-module responses, *N* (%)	*p*-value
Total questions answered	1272	1245^a^	
Participants responding	88	88	
Questions answered correctly	1025 (80.6%)	1163 (93.4%)	< 0.01
Questions answered correctly per participant	11.6 (81%)	13.2 (93.6%)	< 0.01
Knowledge questions			
Question (*Correct answer*)	Pre-module correct answers, (%)	Post-module correct answers, (%)	*p*-value
Which of the following characterizes accurate blood pressure measurement? (*Recording the exact* blood pressure *instead of rounding to the nearest 10*)	83.9%	93.3%	0.04
The automated Omron devices available in JHCP sites have been programmed to measure blood pressure how many times when set in Average mode? (*Three*)	96.7%	100%	0.09
Taking multiple blood pressure readings in one visit leads to a more reliable reading than a single reading, why is this true? (*Taking multiple readings increases the likelihood that the measured value is closest to the true value*)	90%	97.7%	0.03
Which of the following statements about placement of an automated blood pressure cuff is correct? (*Placing the* blood pressure *cuff over a thin shirt sleeve is acceptable*)	46.8%	86.5%	<0.01
The use of a regular adult cuff when a larger one is required would. (*Give a falsely high* blood pressure *reading*)	81.7%	93.3%	0.02
What does the P-set dial found on the automated Omron device do? (*It sets the maximum pressure that the Omron device will inflate to*)	91%	91%	1
Which of the following statements regarding blood pressure measuring equipment is correct? (blood pressure *cuffs should be inspected regularly for leaks*)	82.6%	92%	0.06
An obese patient comes for blood pressure measurement. The extra-large cuff that comes with the automated Omron device is too small for the patient. How should you proceed? (*Obtain a manual* blood pressure *using a thigh cuff instead of using the Omron device*)	67.4%	96.6%	< 0.01
An emergency blood pressure measurement is urgently needed for a sick patient. What should you do? (*Take a single* blood pressure *reading using the automated Omron device*)	94.4%	96.7%	0.47
When using the Omron HEM-907XL, which of the following values is already set when it is on Average mode? (*Waiting time until the start of the first measurement*)	74.4%	85.4%	0.07
An automated blood pressure device displays an error code. What should you do next? (*Look at the device and the patient and think stepwise about what might have gone wrong*)	75%	90.9%	< 0.01
How can you position the patient’s arm at the correct level during blood pressure measurement? (*Support the patient’s arm on a flat surface at heart level during measurement*)	95.5%	98.9%	0.17
You return to the room where your patient’s blood pressure is being recorded and find the Omron HEM-907XL device has turned itself off. If the device is not plugged in, how long after the reading is complete does the device turn itself off? (*5 min*)	70.3%	94.3%	< 0.01
A 22 year-old female with no history of hypertension is seeing her doctor for a routine physical. According to JHCP protocol, how should you measure her blood pressure? (*Take an average reading using the automated Omron device*)	80.2%	91%	0.04
Attitudes questions			
Question	Pre-module response 1 = strongly disagree 5 = strongly agree (mean)	Post-module response 1 = strongly disagree 5 = strongly agree (mean)	*p*-value
Overall	4.2	4.3	0.33
Taking blood pressure with an automated device saves time.	3.4	3.4	0.98
Using the “AVG” (average) mode on the Omron device is only necessary for patients with high blood pressure.	4	4.3	0.19
When using an automated device to measure blood pressure, patient positioning is not important.	4.7	4.7	0.90
Following the blood pressure measurement protocol will result in better management of hypertension for patients.	4.7	4.7	0.90
Allowing patients to rest at least 3 min before taking a blood pressure measurement will result in a more accurate blood pressure reading.	4.5	4.5	0.50

^a^Fewer post-module responses were recorded due to participants exiting the module early

*JHCP* Johns Hopkins Community Physicians

Greatest improvement in knowledge was seen in questions pertaining to measuring blood pressure in patients wearing long sleeves, handling device errors, turning the device off, measuring blood pressure in obese patients, and selecting the appropriate cuff size.

### Behaviors

Two hundred ten observations were completed, 122 (2.7 observations per CMA) prior to the continuing education program and 88 (2 observations per CMA) subsequently (Table 3). Prior to the educational program, staff adhered to the following elements of the protocol during at least 90% of observations: no vaccine given during blood pressure measurement (100%), no fingerstick during blood pressure measurement (97%), back supported (98%), legs uncrossed (94%), correct cuff size used (97%), used bare arm or thin sleeve (93%), arm at heart level (98%), P-set to auto (98%), patient in position throughout measurement (93%). Following the training program, staff were significantly more likely to explain the protocol to patients (59% pre- vs. 80% post-training, *p* < 0.01), measure an average blood pressure (66% vs. 97%, *p* < 0.01), provide a rest period prior to blood pressure measurement (66% vs. 85%, *p* < 0.01), and record in the electronic medical record (EMR) the average of 3 readings (63% vs. 95%, *p* < 0.01). Following training, staff was less likely to measure blood pressure with the arm at heart level (98% vs. 69%, *p* < 0.01) and use the right arm to measure blood pressure (64% vs 42%, *p* < 0.01); patients were less likely to remain still during cuff inflation (84% vs. 53%, *p* < 0.01).

**Table 3:** Assessment of participants’ blood pressure measurement technique, pre- and post-program (*N* = 210)

Observed behavior	Encounters, *N* (%) Pre-intervention	Encounters, N (%) Post-intervention	*p*-value
Total Observations	122 (58%)	88 (42%)	
Clinical staff behavior			
Explains protocol	72 (59%)	70 (80%)	< 0.01
Does not give vaccine during blood pressure measurement	122 (100%)	88 (100%)	1
Does not do fingerstick during blood pressure measurement	118 (97%)	85 (97%)	.98
Positions patient with back supported	119 (98%)	87 (99%)	.76
Positions patient with legs uncrossed	114 (94%)	87 (99%)	.08
Uses correct cuff size	117 (97%)	82 (93%)	.24
Palpates brachial artery	16 (13%)	7 (8%)	.22
Measures blood pressure in right arm	77 (64%)	37 (42%)	< 0.01
Applies cuff to bare arm or over thin sleeve	113 (93%)	85 (97%)	.31
Positions patient with arm supported	107 (88%)	67 (76%)	.02
Positions patient with arm at heart level	119 (98%)	61 (69%)	< 0.01
Uses device with P-set on auto mode	117 (98%)	87 (99%)	.75
Uses device in Average mode	78 (66%)	85 (97%)	< 0.01
Provides patient with a rest period	80 (66%)	75 (85%)	< 0.01
Records average of 3 blood pressure readings	76 (63%)	84 (95%)	< 0.01
Patient behaviors			
Patient remains in position throughout measurement	112 (93%)	86 (98%)	.1
Patient remains quiet during cuff inflation	94 (78%)	75 (85%)	.17
Patient remains still during cuff inflation	102 (84%)	47 (53%)	< 0.01

## Discussion

Provider and staff education are a critical component of implementing and institutionalizing quality improvement programs. Health system leaders must disseminate information about new initiatives, protocols, and techniques effectively in order to ensure that clinicians and staff members provide standardized care across settings [18]. Major barriers to delivering effective training include the logistical difficulties and expense associated with maintaining an adequate pool of educators, coordinating training sessions with providers’ clinical schedules, standardizing training across sites, and ensuring ongoing training for staff. Online educational platforms can help organizations address these gaps. However, little is known about how these tools can facilitate adoption, implementation, and sustainability of quality improvement initiatives [10, 16].

This study highlights important features of online education through the perspective of a program designed to improve blood pressure measurement. Knowledge of correct blood pressure measurement technique improved significantly among both medical assistants and nurses at these practices following a brief online continuing education program. Direct observation data revealed that following completion of the program clinical staff was significantly more likely to explain the protocol to patients, provide a rest period prior to blood pressure measurement, and measure and record the average blood pressure, but less likely to measure blood pressure with the arm at heart level and to use the right arm to measure blood pressure.

Sustainable healthcare interventions tend to be those that have a defining mission, are well integrated into the organizational culture, have an institutional champion, are adaptable, and can be conducted without a strain on existing resources [22, 23]. Our program had a succinct goal, involved senior leadership in the organization, was integrated with existing training structure at the time of hire as well as ongoing training, and included a platform for rapid dissemination without a strain on resources [11]. The educational modules and the implementation processes are modifiable when an issue arises such as a wrong answer or new medical information. Since adoption, this program has been used annually for 4 years to train and retrain staff at the six sites, demonstrating the routinisation of the initiative.

We found baseline knowledge gaps in basic blood pressure measurement among active clinical staff, even though all had been deemed proficient in the ReD CHiP program’s techniques at program implementation or at the time of hire (if they were employed after implementation). This is consistent with prior literature demonstrating knowledge gaps among outpatient staff [1, 2]. The greatest improvement in knowledge was seen for items modeled using multiple modalities in the educational program, including verbal, text, and visual cues: patient positioning, selecting the appropriate cuff size, and explaining the process to the patient.

The training program did not improve attitudes towards accurate blood pressure measurement. Baseline attitudes toward accurate blood pressure measurement were quite high, perhaps due to ongoing training associated with the quality improvement project. Formats other than online training may be more effective in changing attitudes toward effective blood pressure measurement. Awareness of organization and site-level barriers and tailoring interventions to address these obstacles may be one way to change attitudes and behaviors of clinical staff [24].

Direct observation data revealed that completing the training program was associated with improvement in certain steps in the blood pressure protocol, including explaining the protocol to patients, providing a rest period, use of average mode, and recording the average reading in the EMR. Several of these were linked behaviors, as the use of average mode on the device included a rest period and calculated the average blood pressure for recording in the EMR. Subsequent to the training, staff was less likely to use the right arm for blood pressure measurement and to support the arm at heart level. This latter point may be due to the incorrect response recorded during the pre-assessment. Failure to emphasize this information in the training program may also be responsible. Studies of both online and in-person training programs have described a decrement in immediate post-program knowledge gains back to pre-training values several months following the program, indicating a role for supervised practice to promote knowledge retention [25, 26].

Limitations to this study include short follow-up time to assess knowledge, attitudinal, and behavioral outcomes, and a single health system in one specialty. We do not know the generalisability of our findings to other specialties or practices, nor whether knowledge gains were maintained among staff. As this training program was purely an educational intervention and did not address other potential barriers to blood pressure control such as knowledge of guidelines and clinical inertia, we do not know whether improvements in knowledge and behaviors will translate to improved blood pressure control. Prior studies have shown that adherence to AHA blood pressure guidelines impacts treatment decisions as well as measured blood pressure values [2]. The Hawthorne effect is a concern given any direct observation data, which we tried to mitigate by not revealing to clinical staff that the blood pressure measurement process was being observed. Different observers conducted the pre- and post-program observations, which may have caused measurement bias, which we tried to minimize through a rigorous training program and quality control data in mock observations.

## Conclusions

In conclusion, a brief online continuing education program, integrated into an existing organizational training structure, was associated with improvements in knowledge of correct blood pressure measurement techniques, explaining behaviors to patients, and taking multiple measurements using an automated blood pressure device among medical assistants and nurses at six practice sites. To our knowledge, this is the first published study to address use of an online educational program towards sustainable quality improvement across a health system. Such training holds the promise of promoting sustainability of education that requires ongoing reinforcement or knowledge. However, improving attitudes and skills may require a multimodal approach, including a combination of web-based and live training tailored to the existing organizational culture.

## Additional file

Additional file 1: Pre- and post-intervention surveys are the questionnaires used to assess knowledge and attitudes of clinical staff before and following completion of the continuing education program. (DOCX 3714 kb)
Additional file 2: Observation Form is the form used by RAs to record demographic information and observations about blood pressure measurement during the intake process. (DOCX 17 kb)
Additional file 3: Knowledge assessment data is the deidentified dataset containing the results of our pre- and post-module knowledge assessment. (XLS 441 kb)

## Abbreviations

AHA: American heart association
CMA: Certified medical assistant
EMR: Electronic medical record
JHCP: Johns Hopkins Community Physicians
LPN: Licensed practical nurse
Project ReD CHiP: Reducing disparities and controlling hypertension in primary care
RA: Research assistant
RN: Registered nurse
