# Supplementary material for: Promoting sustainability in quality improvement: an evaluation of a web-based continuing education program in blood pressure measurement
Source: BMC Fam Pract. 2018 Jan 10;19:13. doi: 10.1186/s12875-017-0682-5 (PMC5761193; doi:10.1186/s12875-017-0682-5)
Supplement: Additional file 1: — Pre- and post-intervention surveys are the questionnaires used to assess knowledge and attitudes of clinical staff before and following completion of the continuing education program. (DOCX 3714 kb) [file 12875_2017_682_MOESM1_ESM.docx]

# Blood Pressure Management Training (Part 1)

**Quiz Settings**

| **Property** | **Setting** |
| --- | --- |
| Passing Score | 0% |
| Display Point Value | No |
| Randomize Questions |  |
| Pre Attitudes Questions | No |
| Pre Knowledge Questions | No |
| Total Number of Questions | 16 |
| Total Number of Questions to Ask | All |
| Display User Score | No |
| Display Passing Score | No |
| Display Pass Messages | No |
| Display Fail Messages | Yes |
| Pass Email recipient | No |
| Fail Email recipient | No |

# Questions

Pre Attitudes Questions

**1. Please rate the following statements about the automated blood pressure machines (Omron devices) and the blood pressure measurement protocol. Your answers to these questions are confidential and will not affect your final score.**

*(Likert Scale Question, 0 points, 1 attempt permitted)*


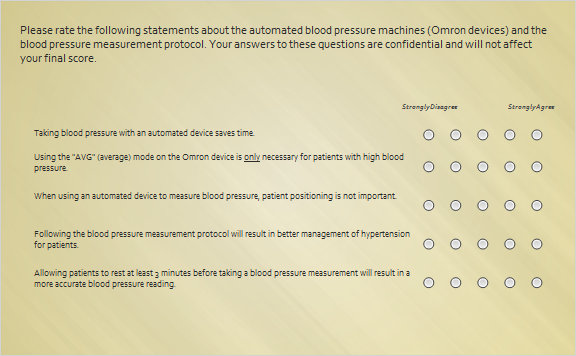


| **Statement** | **Strongly Disagree** | **Somewhat Disagree** | **Neutral** | **Somewhat Agree** | **Strongly Agree** |
| --- | --- | --- | --- | --- | --- |
| Taking blood pressure with an automated device saves time. |  |  |  |  |  |
| Using the "AVG" (average) mode on the Omron device is only necessary for patients with high blood pressure. |  |  |  |  |  |
| When using an automated device to measure blood pressure, patient positioning is not important. |  |  |  |  |  |
| Following the blood pressure measurement protocol will result in better management of hypertension for patients. |  |  |  |  |  |
| Allowing patients to rest at least 3 minutes before taking a blood pressure measurement will result in a more accurate blood pressure reading. |  |  |  |  |  |

Pre Knowlege Questions

**1. Which of the following characterizes accurate blood pressure measurement?**

*(Multiple Choice Question, 10 points, 1 attempt permitted)*


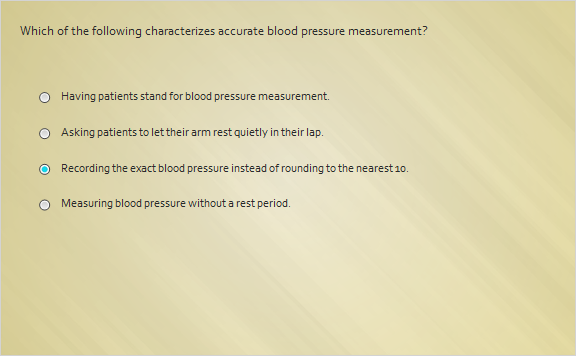


| **Correct** | **Choice** |
| --- | --- |
|  | Having patients stand for blood pressure measurement. |
|  | Asking patients to let their arm rest quietly in their lap. |
| X | Recording the exact blood pressure instead of rounding to the nearest 10. |
|  | Measuring blood pressure without a rest period. |

**2. The automated Omron devices available in JHCP sites have been programmed to measure blood pressure how many times when set on the AVG (average) mode?**

*(Multiple Choice Question, 10 points, 1 attempt permitted)*


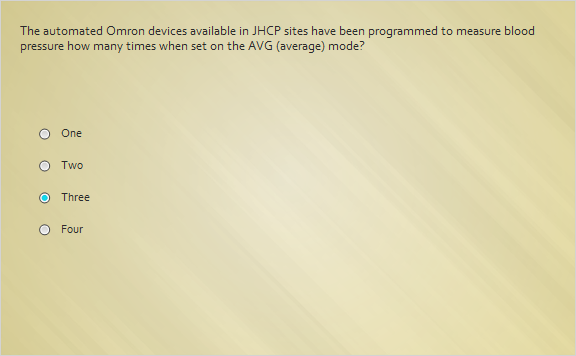


| **Correct** | **Choice** |
| --- | --- |
|  | One |
|  | Two |
| X | Three |
|  | Four |

**3. Taking multiple blood pressure readings in one visit leads to a more reliable reading than a single reading. Why is this true?**

*(Multiple Choice Question, 10 points, 1 attempt permitted)*


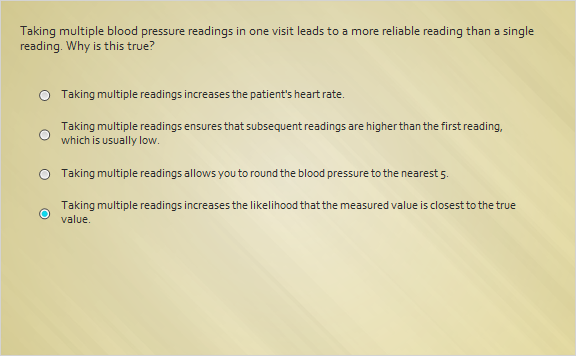


| **Correct** | **Choice** |
| --- | --- |
|  | Taking multiple readings increases the patient's heart rate. |
|  | Taking multiple readings ensures that subsequent readings are higher than the first reading, which is usually low. |
|  | Taking multiple readings allows you to round the blood pressure to the nearest 5. |
| X | Taking multiple readings increases the likelihood that the measured value is closest to the true value. |

***4. Which of the following statements regarding patient positioning for blood pressure measurement is correct?**

*(Multiple Choice Question, 10 points, 1 attempt permitted)*


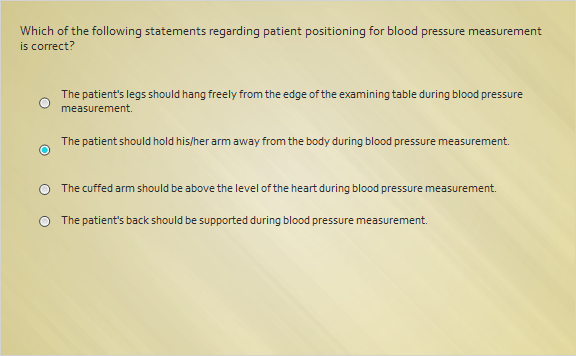


| **Correct** | **Choice** |
| --- | --- |
|  | The patient's legs should hang freely from the edge of the examining table during blood pressure measurement. |
| X | The patient should hold his/her arm away from the body during blood pressure measurement. |
|  | The cuffed arm should be above the level of the heart during blood pressure measurement. |
|  | The patient's back should be supported during blood pressure measurement. |

**5. Which of the following statements about placement of an automated blood pressure cuff is correct?**

*(Multiple Choice Question, 10 points, 1 attempt permitted)*


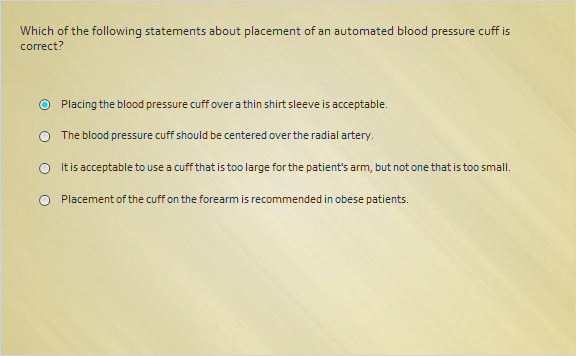


| **Correct** | **Choice** |
| --- | --- |
| X | Placing the blood pressure cuff over a thin shirt sleeve is acceptable. |
|  | The blood pressure cuff should be centered over the radial artery. |
|  | It is acceptable to use a cuff that is too large for the patient's arm, but not one that is too small. |
|  | Placement of the cuff on the forearm is recommended in obese patients. |

**6. The use of a regular adult cuff when a larger one is required would**

*(Multiple Choice Question, 10 points, 1 attempt permitted)*


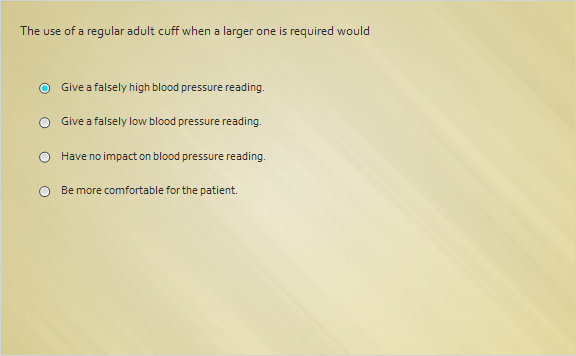


| **Correct** | **Choice** |
| --- | --- |
| X | Give a falsely high blood pressure reading. |
|  | Give a falsely low blood pressure reading. |
|  | Have no impact on blood pressure reading. |
|  | Be more comfortable for the patient. |

**7. What does the P-Set dial found on the automated Omron device do?**

*(Multiple Choice Question, 10 points, 1 attempt permitted)*


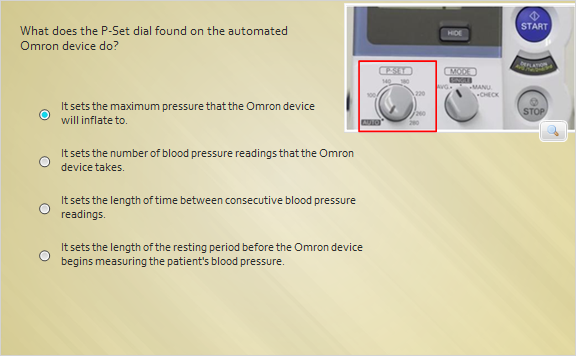


| **Correct** | **Choice** |
| --- | --- |
| X | It sets the maximum pressure that the Omron device will inflate to. |
|  | It sets the number of blood pressure readings that the Omron device takes. |
|  | It sets the length of time between consecutive blood pressure readings. |
|  | It sets the length of the resting period before the Omron device begins measuring the patient's blood pressure. |

**8. Which of the following statements regarding blood pressure measuring equipment is correct?**

*(Multiple Choice Question, 10 points, 1 attempt permitted)*


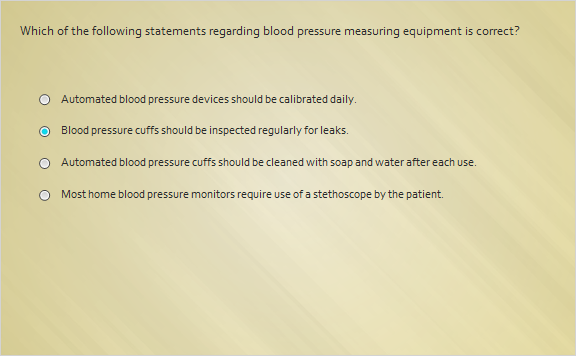


| **Correct** | **Choice** |
| --- | --- |
|  | Automated blood pressure devices should be calibrated daily. |
| X | Blood pressure cuffs should be inspected regularly for leaks. |
|  | Automated blood pressure cuffs should be cleaned with soap and water after each use. |
|  | Most home blood pressure monitors require use of a stethoscope by the patient. |

**9. An obese patient comes for blood pressure measurement. The extra-large (XL) cuff that comes with the automated Omron device is too small for the patient. How should you proceed?**

*(Multiple Choice Question, 10 points, 1 attempt permitted)*


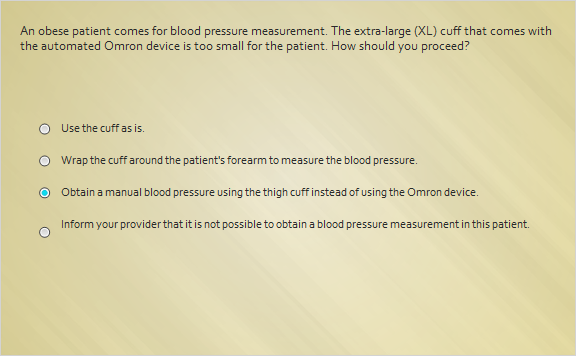


| **Correct** | **Choice** |
| --- | --- |
|  | Use the cuff as is. |
|  | Wrap the cuff around the patient's forearm to measure the blood pressure. |
| X | Obtain a manual blood pressure using the thigh cuff instead of using the Omron device. |
|  | Inform your provider that it is not possible to obtain a blood pressure measurement in this patient. |

**10. An emergency blood pressure measurement is urgently needed for a sick patient.**

**What should you do?**

*(Multiple Choice Question, 10 points, 1 attempt permitted)*


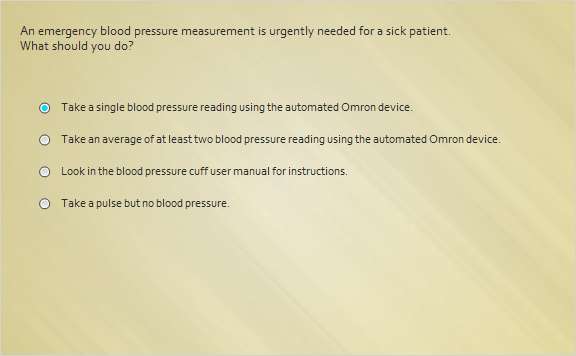


| **Correct** | **Choice** |
| --- | --- |
| X | Take a single blood pressure reading using the automated Omron device. |
|  | Take an average of at least two blood pressure reading using the automated Omron device. |
|  | Look in the blood pressure cuff user manual for instructions. |
|  | Take a pulse but no blood pressure. |

**11. When using the Omron HEM-907XL, which of the following values is already set when it is on AVG (average) mode?**

*(Multiple Choice Question, 10 points, 1 attempt permitted)*


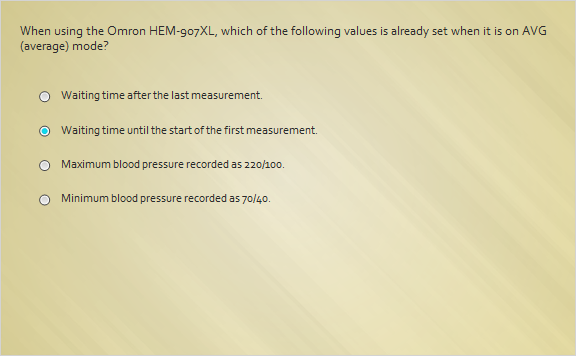


| **Correct** | **Choice** |
| --- | --- |
|  | Waiting time after the last measurement. |
| X | Waiting time until the start of the first measurement. |
|  | Maximum blood pressure recorded as 220/100. |
|  | Minimum blood pressure recorded as 70/40. |

**12. An automated blood pressure device displays an error code. What should you do next?**

*(Multiple Choice Question, 10 points, 1 attempt permitted)*


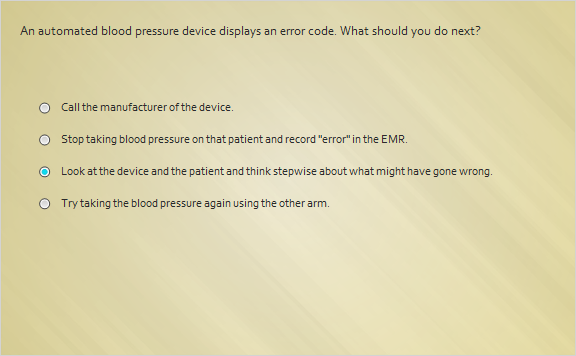


| **Correct** | **Choice** |
| --- | --- |
|  | Call the manufacturer of the device. |
|  | Stop taking blood pressure on that patient and record "error" in the EMR. |
| X | Look at the device and the patient and think stepwise about what might have gone wrong. |
|  | Try taking the blood pressure again using the other arm. |

**13. How can you position the patient's arm at the correct level during blood pressure measurement?**

*(Multiple Choice Question, 10 points, 1 attempt permitted)*


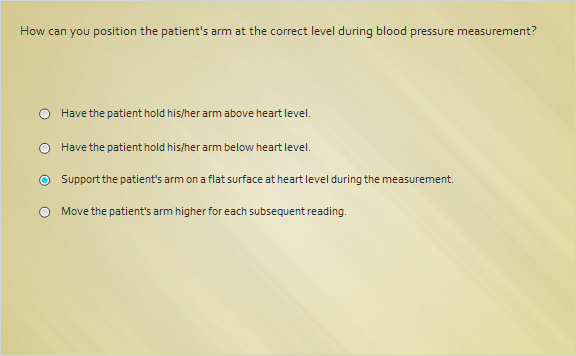


| **Correct** | **Choice** |
| --- | --- |
|  | Have the patient hold his/her arm above heart level. |
|  | Have the patient hold his/her arm below heart level. |
| X | Support the patient's arm on a flat surface at heart level during the measurement. |
|  | Move the patient's arm higher for each subsequent reading. |

**14. You return to the room where your patient's blood pressure is being recorded and find the Omron HEM-907XL device has turned itself off. If the device is not plugged in, how long after the reading is complete does the device turn itself off?**

*(Multiple Choice Question, 10 points, 1 attempt permitted)*


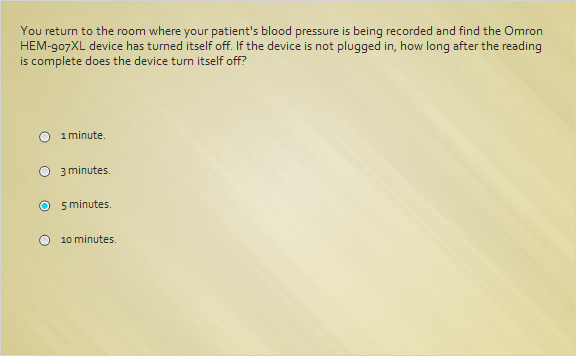


| **Correct** | **Choice** |
| --- | --- |
|  | 1 minute. |
|  | 3 minutes. |
| X | 5 minutes. |
|  | 10 minutes. |

**15. A 22-year-old female with no history of hypertension is seeing her doctor for a routine physical. According to JHCP protocol, how should you measure her blood pressure?**

*(Multiple Choice Question, 10 points, 1 attempt permitted)*


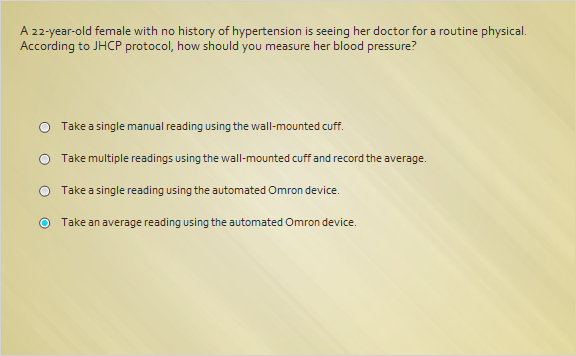


| **Correct** | **Choice** |
| --- | --- |
|  | Take a single manual reading using the wall-mounted cuff. |
|  | Take multiple readings using the wall-mounted cuff and record the average. |
|  | Take a single reading using the automated Omron device. |
| X | Take an average reading using the automated Omron device. |

# Blood Pressure Management Training (Part 2)

**Quiz Settings**

| **Property** | **Setting** |
| --- | --- |
| Passing Score | 80% |
| Display Point Value | No |
| Randomize Questions |  |
| Post Attitudes Questions | No |
| Post Knowlege Questions | No |
| Demographic Questions | No |
| Total Number of Questions | 23 |
| Total Number of Questions to Ask | All |
| Display User Score | No |
| Display Passing Score | No |
| Display Pass Messages | Yes |
| Display Fail Messages | Yes |
| Pass Email recipient | No |
| Fail Email recipient | No |

# Questions

Post Attitudes Questions

**1. Please rate the following statements about the automated blood pressure machines (Omron devices) and the blood pressure measurement protocol. Your answers to these questions are confidential and will not affect your final score.**

*(Likert Scale Question, 0 points, 1 attempt permitted)*


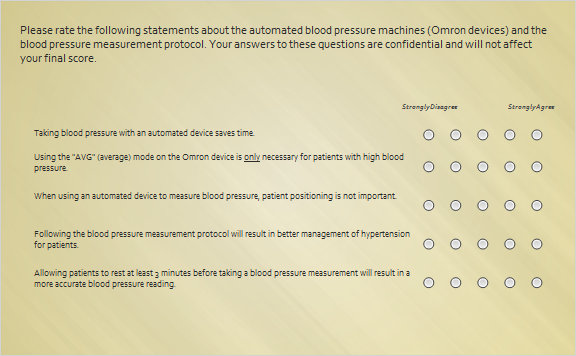


| **Statement** | **Strongly Disagree** | **Somewhat Disagree** | **Neutral** | **Somewhat Agree** | **Strongly Agree** |
| --- | --- | --- | --- | --- | --- |
| Taking blood pressure with an automated device saves time. |  |  |  |  |  |
| Using the "AVG" (average) mode on the Omron device is only necessary for patients with high blood pressure. |  |  |  |  |  |
| When using an automated device to measure blood pressure, patient positioning is not important. |  |  |  |  |  |
| Following the blood pressure measurement protocol will result in better management of hypertension for patients. |  |  |  |  |  |
| Allowing patients to rest at least 3 minutes before taking a blood pressure measurement will result in a more accurate blood pressure reading. |  |  |  |  |  |

Feedback:

Post Knowledge Questions

**1. Which of the following characterizes accurate blood pressure measurement?**

*(Multiple Choice Question, 10 points, 1 attempt permitted)*


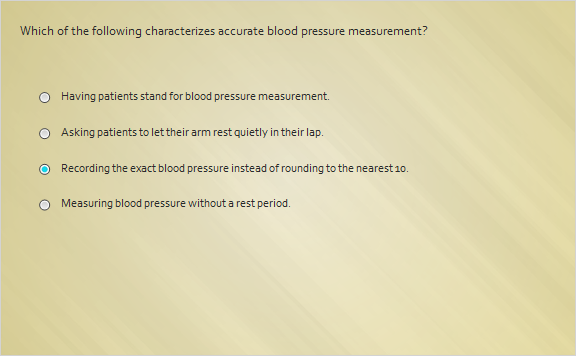


| **Correct** | **Choice** |
| --- | --- |
|  | Having patients stand for blood pressure measurement. |
|  | Asking patients to let their arm rest quietly in their lap. |
| X | Recording the exact blood pressure instead of rounding to the nearest 10. |
|  | Measuring blood pressure without a rest period. |

Feedback when correct: That's right! You selected the correct response.

Feedback when incorrect: You did not select the correct response.

**2. The automated Omron devices available in JHCP sites have been programmed to measure blood pressure how many times when set on the AVG (average) mode?**

*(Multiple Choice Question, 10 points, 1 attempt permitted)*


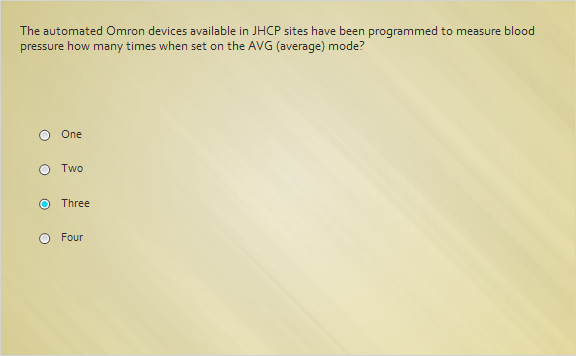


| **Correct** | **Choice** |
| --- | --- |
|  | One |
|  | Two |
| X | Three |
|  | Four |

Feedback when correct: That's right! You selected the correct response.

Feedback when incorrect: You did not select the correct response.

**3. Taking multiple blood pressure readings in one visit leads to a more reliable reading than a single reading. Why is this true?**

*(Multiple Choice Question, 10 points, 1 attempt permitted)*


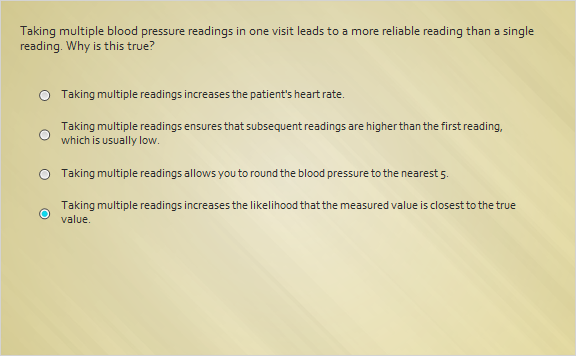


| **Correct** | **Choice** |
| --- | --- |
|  | Taking multiple readings increases the patient's heart rate. |
|  | Taking multiple readings ensures that subsequent readings are higher than the first reading, which is usually low. |
|  | Taking multiple readings allows you to round the blood pressure to the nearest 5. |
| X | Taking multiple readings increases the likelihood that the measured value is closest to the true value. |

Feedback when correct: That's right! You selected the correct response.

Feedback when incorrect: You did not select the correct response.

**4. Which of the following statements regarding patient positioning for blood pressure measurement is correct?**

*(Multiple Choice Question, 10 points, 1 attempt permitted)*


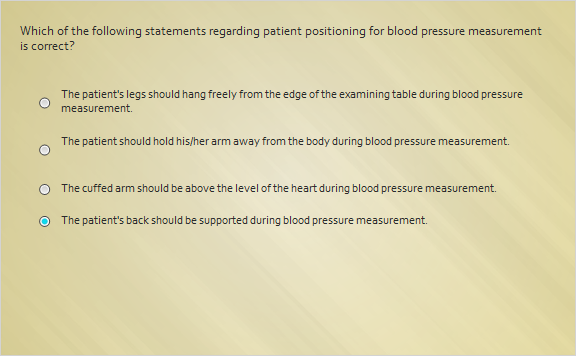


| **Correct** | **Choice** |
| --- | --- |
|  | The patient's legs should hang freely from the edge of the examining table during blood pressure measurement. |
|  | The patient should hold his/her arm away from the body during blood pressure measurement. |
|  | The cuffed arm should be above the level of the heart during blood pressure measurement. |
| X | The patient's back should be supported during blood pressure measurement. |

Feedback when correct: That's right! You selected the correct response.

Feedback when incorrect: You did not select the correct response.

**5. Which of the following statements about placement of an automated blood pressure cuff is correct?**

*(Multiple Choice Question, 10 points, 1 attempt permitted)*


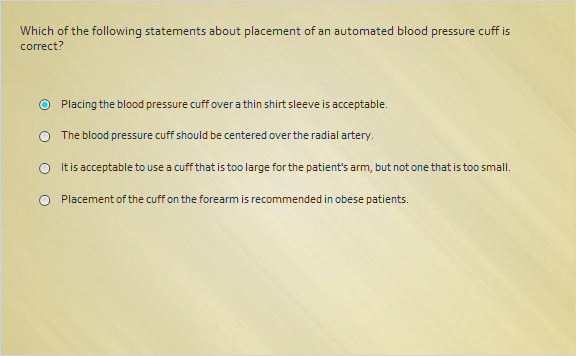


| **Correct** | **Choice** |
| --- | --- |
| X | Placing the blood pressure cuff over a thin shirt sleeve is acceptable. |
|  | The blood pressure cuff should be centered over the radial artery. |
|  | It is acceptable to use a cuff that is too large for the patient's arm, but not one that is too small. |
|  | Placement of the cuff on the forearm is recommended in obese patients. |

Feedback when correct: That's right! You selected the correct response.

Feedback when incorrect: You did not select the correct response.

**6. The use of a regular adult cuff when a larger one is required would**

*(Multiple Choice Question, 10 points, 1 attempt permitted)*


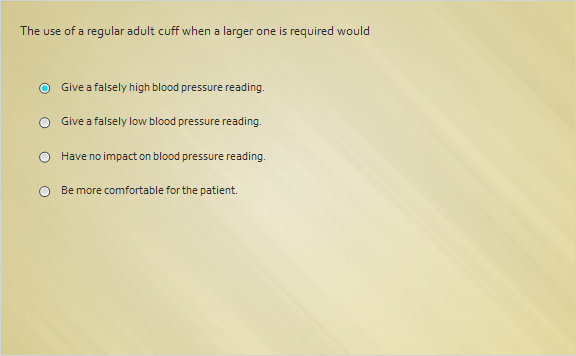


| **Correct** | **Choice** |
| --- | --- |
| X | Give a falsely high blood pressure reading. |
|  | Give a falsely low blood pressure reading. |
|  | Have no impact on blood pressure reading. |
|  | Be more comfortable for the patient. |

Feedback when correct: That's right! You selected the correct response.

Feedback when incorrect: You did not select the correct response.

**7. What does the P-Set dial found on the automated Omron device do?**

*(Multiple Choice Question, 10 points, 1 attempt permitted)*


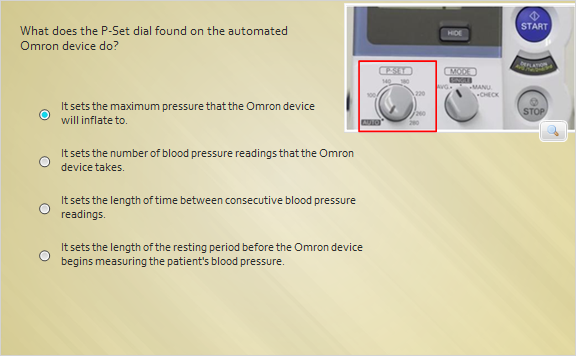


| **Correct** | **Choice** |
| --- | --- |
| X | It sets the maximum pressure that the Omron device will inflate to. |
|  | It sets the number of blood pressure readings that the Omron device takes. |
|  | It sets the length of time between consecutive blood pressure readings. |
|  | It sets the length of the resting period before the Omron device begins measuring the patient's blood pressure. |

Feedback when correct: That's right! You selected the correct response.

Feedback when incorrect: You did not select the correct response.

**8. Which of the following statements regarding blood pressure measuring equipment is correct?**

*(Multiple Choice Question, 10 points, 1 attempt permitted)*


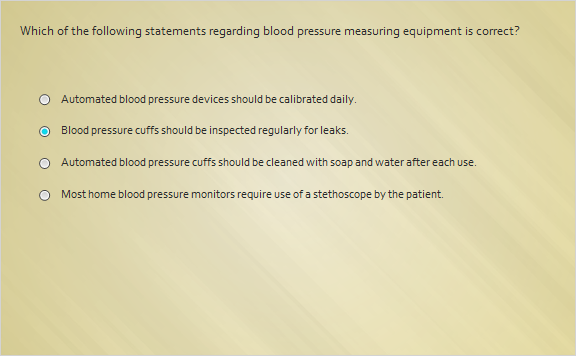


| **Correct** | **Choice** |
| --- | --- |
|  | Automated blood pressure devices should be calibrated daily. |
| X | Blood pressure cuffs should be inspected regularly for leaks. |
|  | Automated blood pressure cuffs should be cleaned with soap and water after each use. |
|  | Most home blood pressure monitors require use of a stethoscope by the patient. |

Feedback when correct: That's right! You selected the correct response.

Feedback when incorrect: You did not select the correct response.

**9. An obese patient comes for blood pressure measurement. The extra-large (XL) cuff that comes with the automated Omron device is too small for the patient. How should you proceed?**

*(Multiple Choice Question, 10 points, 1 attempt permitted)*


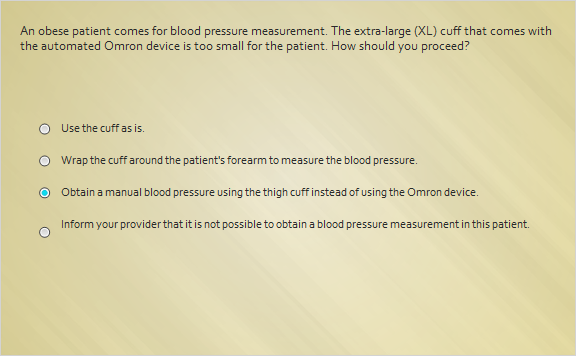


| **Correct** | **Choice** |
| --- | --- |
|  | Use the cuff as is. |
|  | Wrap the cuff around the patient's forearm to measure the blood pressure. |
| X | Obtain a manual blood pressure using the thigh cuff instead of using the Omron device. |
|  | Inform your provider that it is not possible to obtain a blood pressure measurement in this patient. |

Feedback when correct: That's right! You selected the correct response.

Feedback when incorrect: You did not select the correct response.

**10. An emergency blood pressure measurement is urgently needed for a sick patient. What should you do?**

*(Multiple Choice Question, 10 points, 1 attempt permitted)*


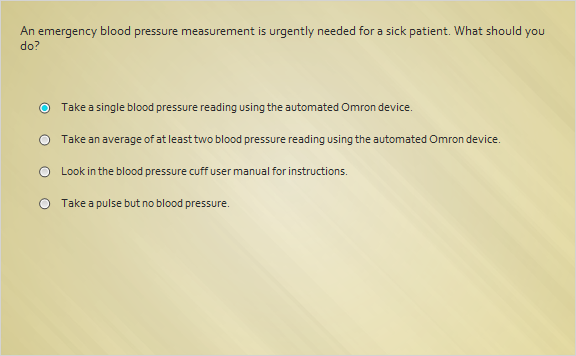


| **Correct** | **Choice** |
| --- | --- |
| X | Take a single blood pressure reading using the automated Omron device. |
|  | Take an average of at least two blood pressure reading using the automated Omron device. |
|  | Look in the blood pressure cuff user manual for instructions. |
|  | Take a pulse but no blood pressure. |

Feedback when correct: That's right! You selected the correct response.

Feedback when incorrect: You did not select the correct response.

**11. When using the Omron HEM-907XL, which of the following values is already set when it is on AVG (average) mode?**

*(Multiple Choice Question, 10 points, 1 attempt permitted)*


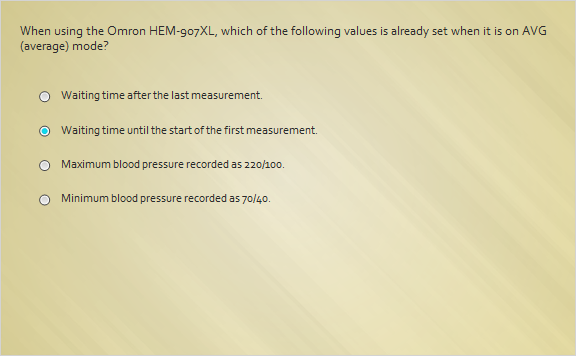


| **Correct** | **Choice** |
| --- | --- |
|  | Waiting time after the last measurement. |
| X | Waiting time until the start of the first measurement. |
|  | Maximum blood pressure recorded as 220/100. |
|  | Minimum blood pressure recorded as 70/40. |

Feedback when correct: That's right! You selected the correct response.

Feedback when incorrect: You did not select the correct response.

**12. An automated blood pressure device displays an error code. What should you do next?**

*(Multiple Choice Question, 10 points, 1 attempt permitted)*


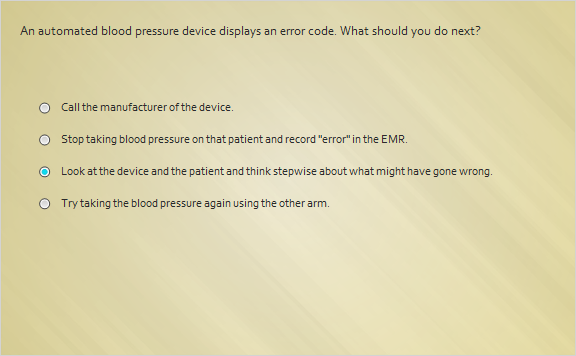


| **Correct** | **Choice** |
| --- | --- |
|  | Call the manufacturer of the device. |
|  | Stop taking blood pressure on that patient and record "error" in the EMR. |
| X | Look at the device and the patient and think stepwise about what might have gone wrong. |
|  | Try taking the blood pressure again using the other arm. |

Feedback when correct: That's right! You selected the correct response.

Feedback when incorrect: You did not select the correct response.

**13. How can you position the patient's arm at the correct level during blood pressure measurement?**

*(Multiple Choice Question, 10 points, 1 attempt permitted)*


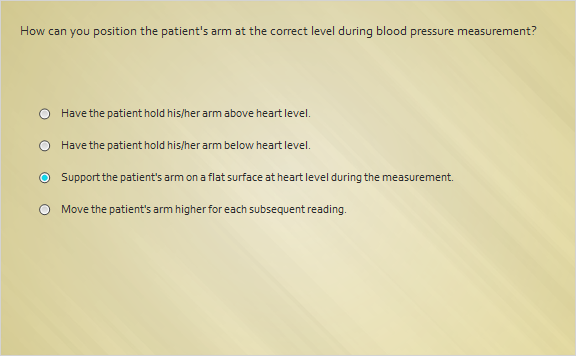


| **Correct** | **Choice** |
| --- | --- |
|  | Have the patient hold his/her arm above heart level. |
|  | Have the patient hold his/her arm below heart level. |
| X | Support the patient's arm on a flat surface at heart level during the measurement. |
|  | Move the patient's arm higher for each subsequent reading. |

Feedback when correct: That's right! You selected the correct response.

Feedback when incorrect: You did not select the correct response.

**14. You return to the room where your patient's blood pressure is being recorded and find the Omron HEM-907XL device has turned itself off. If the device is not plugged in, how long after the reading is complete does the device turn itself off?**

*(Multiple Choice Question, 10 points, 1 attempt permitted)*


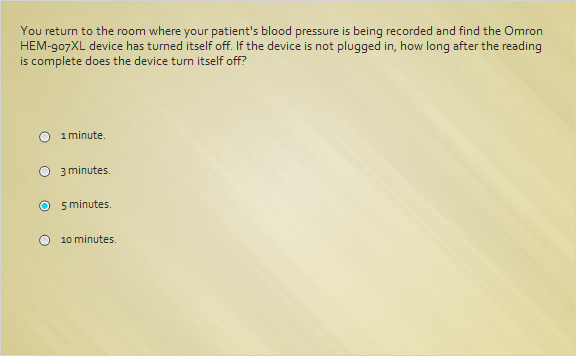


| **Correct** | **Choice** |
| --- | --- |
|  | 1 minute. |
|  | 3 minutes. |
| X | 5 minutes. |
|  | 10 minutes. |

Feedback when correct: That's right! You selected the correct response.

Feedback when incorrect: You did not select the correct response.

**15. A 22-year-old female with no history of hypertension is seeing her doctor for a routine physical. According to JHCP protocol, how should you measure her blood pressure?**

*(Multiple Choice Question, 10 points, 1 attempt permitted)*


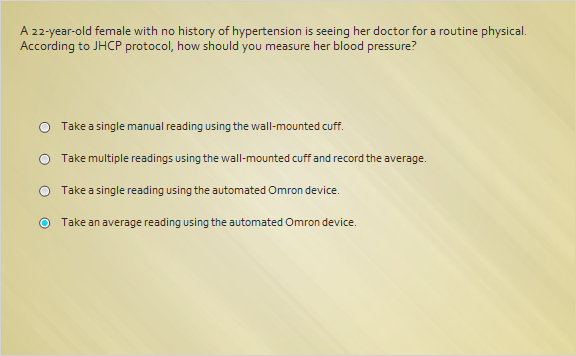


| **Correct** | **Choice** |
| --- | --- |
|  | Take a single manual reading using the wall-mounted cuff. |
|  | Take multiple readings using the wall-mounted cuff and record the average. |
|  | Take a single reading using the automated Omron device. |
| X | Take an average reading using the automated Omron device. |

Feedback when correct: That's right! You selected the correct response.

Feedback when incorrect: You did not select the correct response.

Demographic Questions

**1. What is your age?**

*(Pick One Question, 0 points, 1 attempt permitted)*


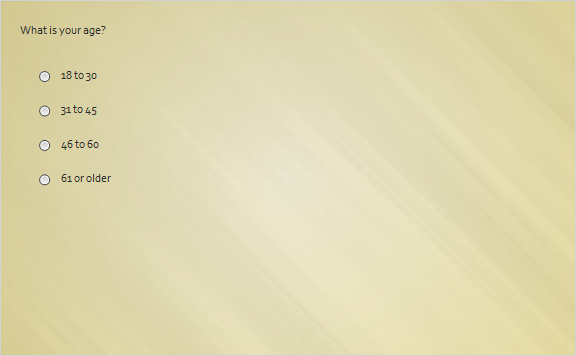


| **Choice** |
| --- |
| 18 to 30 |
| 31 to 45 |
| 46 to 60 |
| 61 or older |

Feedback:

**2. What is your gender?**

*(Pick One Question, 0 points, 1 attempt permitted)*


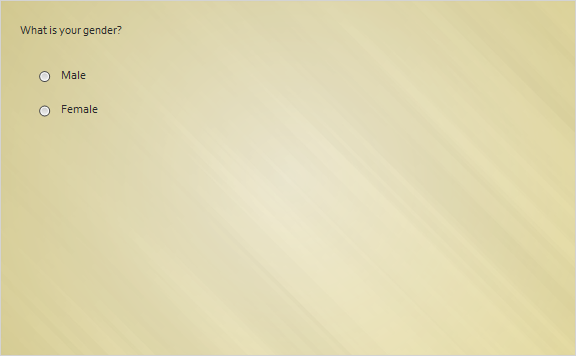


| **Choice** |
| --- |
| Male |
| Female |

Feedback:

**3. Are you of Hispanic, Latino, or Spanish origin?**

*(Pick One Question, 0 points, 1 attempt permitted)*


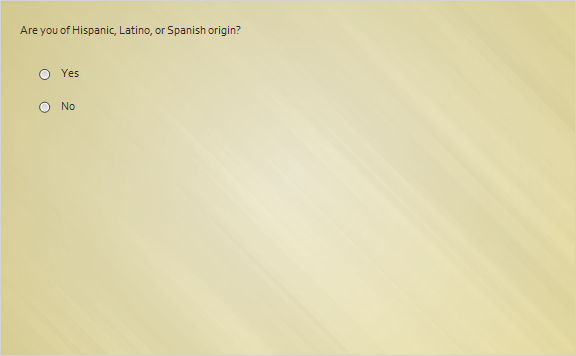


| **Choice** |
| --- |
| Yes |
| No |

Feedback:

**4. What racial group do you belong to? (One or more categories may be selected)**

*(Pick Many Question, 0 points, 1 attempt permitted)*


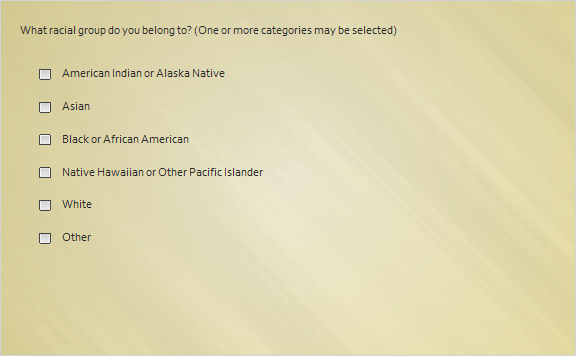


| **Choice** |
| --- |
| American Indian or Alaska Native |
| Asian |
| Black or African American |
| Native Hawaiian or Other Pacific Islander |
| White |
| Other |

Feedback:

**5. What best describes your training?**

*(Pick One Question, 0 points, 1 attempt permitted)*


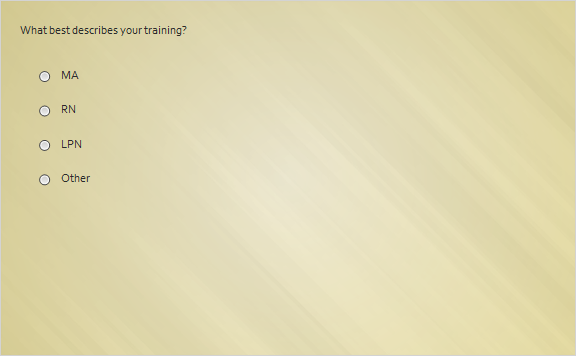


| **Choice** |
| --- |
| MA |
| RN |
| LPN |
| Other |

Feedback:

**6. In which JHCP practice do you work?**

*(Pick Many Question, 0 points, 1 attempt permitted)*


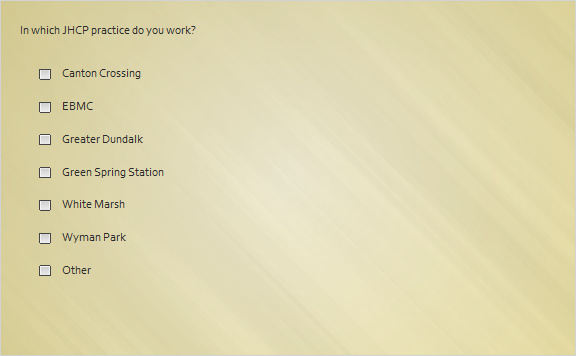


| **Choice** |
| --- |
| Canton Crossing |
| EBMC |
| Greater Dundalk |
| Green Spring Station |
| White Marsh |
| Wyman Park |
| Other |

Feedback:

**7. How long have you been working at your current office/practice location?**

*(Pick One Question, 0 points, 1 attempt permitted)*


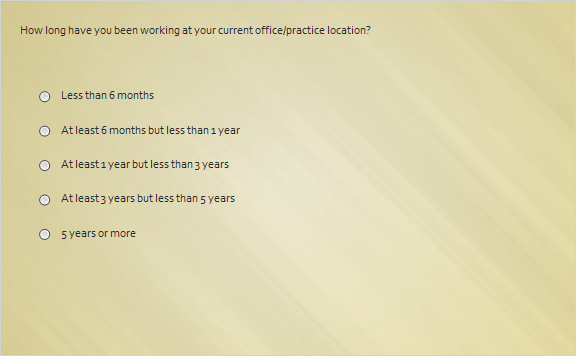


| **Choice** |
| --- |
| Less than 6 months |
| At least 6 months but less than 1 year |
| At least 1 year but less than 3 years |
| At least 3 years but less than 5 years |
| 5 years or more |

Feedback:

*****The pre-test answer to question #4 was incorrectly listed as “The patients should hold his/her arm away from the body during blood pressure measurement.” The correct answer is “The patient’s back should be supported during blood pressure measurement.”
