# Supplementary material for: Promoting sustainability in quality improvement: an evaluation of a web-based continuing education program in blood pressure measurement
Source: BMC Fam Pract. 2018 Jan 10;19:13. doi: 10.1186/s12875-017-0682-5 (PMC5761193; doi:10.1186/s12875-017-0682-5)
Supplement: Additional file 2: — Observation Form is the form used by RAs to record demographic information and observations about blood pressure measurement during the intake process. (DOCX 17 kb) [file 12875_2017_682_MOESM2_ESM.docx]

| **Clinic:** | **Observer:** |
| --- | --- |

**Date:**

**Time Period:** morning afternoon

**Patient**: #_______

**Patient characteristics**

| Age: | <30 years | | | 31-50 years | | >50 years | |
| --- | --- | --- | --- | --- | --- | --- | --- |
|  |  | | |  | |  | |
| Med Hx | HTN | | | DM | | CKD | |
|  |  | | |  | |  | |
| Pt alone? | Yes | | | No | |  | |
|  |  | | |  | |  | |
| Pt ambulates? | Yes | | | No | |  | |
|  | |  | |  | |  | |
| Hispanic/Latino? | | Yes | | No | |  | |
|  | |  | |  | |  | |
| Race | | Am Indian | Asian | | Black/AA | | White |
|  | |  |  | |  | |  |
|  | | Pacific Islander | Multi-racial | | Other | | No response |

**Intake Time**

| **Call from waiting room** | **Arrive to exam room** | **Start intake** | **End vitals** | **End intake** | **Chart to PCP** |
| --- | --- | --- | --- | --- | --- |
| **0 min** |  |  |  |  |  |

**For Manual BP measurements**

| **MA Actions** | | | | | | | |
| --- | --- | --- | --- | --- | --- | --- | --- |
| Back supported | Yes | | | No | | | |
| Feet supported | Yes | | | No | | | |
| Legs are uncrossed | Yes | | | No | | | |
| Cuff size | Correct | | Over-cuff | | | Under-cuff | |
| Wraps cuff on bare arm | Yes | | | No | | | |
| Arm supported | Yes | | | No | | | |
| Cuff at heart level | Correct | | Too high | | | Too low | |
| Rest period | None | | <3 min | | | 3+ min | |
| BP measurements | 1 | 2 | | | 3 | | 4+ |

**Barriers to protocol adherence**

| Error code –  Protocol completed | Error code –  Protocol **not** completed |
| --- | --- |
| Patient arm >52 cm | Protocol does not work with patient |

**Observations**

| **MA Actions** | | | | |
| --- | --- | --- | --- | --- |
| Explains procedure to patient | Yes | | No | |
| Vaccine given after BPM complete | Yes | No | | N/A |
| Fingerstick after BPM complete | Yes | No | | N/A |
| Ensures back supported | Yes | | No | |
| Ensures feet supported | Yes | | No | |
| Ensure legs are uncrossed | Yes | | No | |
| Cuff size | Correct | Over-cuff | | Under-cuff |
| Locates brachial artery by palpation | Yes | | No | |
| Arm selected | Right | Left | | Both |
| Wraps cuff on bare arm/thin sleeve | Yes | | No | |
| Ensures Arm supported | Yes | | No | |
| Ensures cuff at heart level | Correct | Too high | | Too low |
| P-set dial at AUTO | Yes | | No | |
| Mode dial at AVG | Yes | | No | |
| Presses START prior to screening Q | Yes | | No | |
| Patient has full 3 min rest period | Yes | | No | |
| Patient has 3 BP measurements | Yes | | No | |
| Records AVG BP results | Yes | | No | |
| **Patient actions** | | | | |
| Patient in position when cuff in use | Yes | | No | |
| Patient quiet during rest period | Yes | | No | |
| Patient quiet when cuff in use | Yes | | No | |
